# Bioactive Compounds and Aroma Profile of Some Lamiaceae Edible Flowers

**DOI:** 10.3390/plants9060691

**Published:** 2020-05-28

**Authors:** Ilaria Marchioni, Basma Najar, Barbara Ruffoni, Andrea Copetta, Luisa Pistelli, Laura Pistelli

**Affiliations:** 1 Dipartimento di Scienze Agrarie, Alimentari e Agro-Alimentari, Università di Pisa, Via del Borghetto 80, 56124 Pisa, Italy; i.marchioni@studenti.unipi.it (I.M.); laura.pistelli@unipi.it (L.P.); 2 Dipartimento di Farmacia, Università di Pisa, Via Bonanno 6, 56126 Pisa, Italy; luisa.pistelli@unipi.it; 3 CREA—Centro di Ricerca Orticoltura e Florovivaismo, Corso Inglesi 508, 18038 Sanremo, IM, Italy; barbara.ruffoni@crea.gov.it (B.R.); andrea.copetta@crea.gov.it (A.C.); 4 Centro Interdipartimentale di Ricerca “Nutraceutica e Alimentazione per la Salute” (NUTRAFOOD), Università di Pisa, Via del Borghetto 80, 56124 Pisa, Italy

**Keywords:** *Salvia* spp., *Ocimum* spp., *Nepeta* × *faassenii*, *Monarda didyma*, VOCs, nutraceutical properties, essential oil, health effect

## Abstract

Edible flowers are consumed for their appearance, colours, nutritional and healthy properties, but the use is limited by the actual number of the species. Seven edible flowers of the Lamiaceae family (Ocimeae and Mentheae tribes) were investigated: *Monarda didyma* ‘Fireball’, *Nepeta × faassenii* ‘Six Hills Giant’, *Ocimum basilicum* ‘Blue Spice’, *O. basilicum* ‘Cinnamon’, *Ocimum × citriodorum*, *Salvia discolor,* and *Salvia microphylla* ‘Hot Lips’. Total soluble sugars, proteins, polyphenols, carotenoids, ascorbic acid and antioxidant activity were detected. The species of the Mentheae tribe contained higher sugar content than Ocimeae flowers, the opposite with regard to protein content. Ocimeae tribe flowers showed high polyphenols and carotenoids content. The Ocimeae tribe together with two specie of the Mentheae tribe showed an aroma profile dominated by sesquiterpene hydrocarbons (58.0% in *S. discolor* to 77.9% in *Ocimum* × *citriodorum*). Oxygenated monoterpenes prevailed in *Nepeta* and *Monarda,* also present in the essential oil of this latter species (84.5%). By contrast, *Nepeta* and *S. discolor* evidenced non-terpenes as the principal class (41.2% and 77.5%, respectively), while the oxygenated sesquiterpene was the main one in *S. microphylla*. The two varieties of *Ocimum* spp. showed oxygenated monoterpenes as the main class of volatiles.

## 1. Introduction

Lamiaceae (order Lamiales) is a family of flowering species, also known as the mint family [1]. The taxonomy rank is composed of 236 genera and 6900–7200 species, distributed all over the world [2]. Lamiaceae is divided in 12 subfamilies [3] of which Nepetoidae is one of the most clearly defined [4] and has strongly aromatic species with volatile terpenoids [1].

Lamiaceae are usually herbs, subshrubs, or shrubs. Their leaves and flowers are generally scented, and this is a distinctive feature of this family. Many Lamiaceae species produce a wide spectrum of bioactive compounds (flavonoids, terpenoids, phenolics and alkaloids), that are characterized by numerous biological activities (e.g., antioxidant, anti-inflammatory, and antibacterial properties) [5,6,7,8,9]. Therefore, several species were listed in the official Pharmacopoeias [10] and currently used in pharmaceutical, cosmetic, food and pesticides industries [11]. Furthermore, many members of the Lamiaceae family are widely cultivated as culinary herbs, such as basil, oregano, rosemary, thyme, mint, and sage [12]. Most of them produce edible flowers [13], even if their consumption is lower compared to that of the leaves, generally used as seasonings.

Edible flowers are consumed in different part of the world, since they are able to improve appearance, colour and nutritive values of meals [13,14,15]. Although Lu et al. [16] reported that 180 species, 100 genera and 97 families produce edible flowers, no official list has been published by any international organization [13,17] and only a small part of them have been studied so far [18]. Several scientific reports highlight their nutritional and healthy properties [18,19,20]. In fact, even though edible flowers are usually composed of 70% to 95% of water [18], pollen, nectar and petals can be a real source of primary metabolites [13,21], vitamins [22], and minerals [23,24]. Edible flowers are also rich in antioxidant molecules (e.g., polyphenols and pigments), useful to prevent several diseases [16,18]. Aroma and scent are further distinctive features of most of the edible flowers currently consumed [17,25]. Both are essential to entice people to purchase this product [26].

In order to improve the research on edible flowers, plants of 2 tribes of the subfamily Nepetoideae were investigated herein: three types of *O. basilicum* (Ob) belonging to the tribe Ocimeae and four different species of the Mentheae tribe. The *Ocimum* genus, with its 150 species, is widely distributed in the temperate region of the world [27,28]. Various cultivars differ in flowers’ and leaves’ morphology (colour size, shape), as well as the composition of substances like essential oils (EOs) [29]. *O. basilicum* var. *italicum*, also called Sweet Basil, is cultivated all over the world for its EO as well as being a culinary and ornamental plant [28]. EOs of *O. basilicum* have important biological activities and depending on environmental conditions, age of plant, agronomic techniques, and their chemotypes [29,30]. The chemotypes are based on 1–2 predominant constituents of leaf EO. According to the literature, the studied types *O. basilicum* ‘Blue Spice’ (Ob-BS), *O. basilicum* ‘Cinnamon’ (Ob-Cn) and *O.* × *citriodorum* (Ob-Ct) belong to three different chemotypes [29].

The first Menthae member includes one of the most popular genera and presumably the largest and widely distributed one within the Lamiaceae family: *Salvia* [31,32]. Beside their ethnobotanical importance, plants of these taxa have a commercial importance due to their culinary, nutraceutical, medicinal and fragrance uses. Within the genus, *S. discolor* (S. disc) and *S. microphylla* (S. micro) are plants initially used for ornamental purpose [33]; they are of a good nutritive intake [34] and are known for their aromatic volatile compounds and medicinal properties [35,36,37]. *Nepeta* with its 280 spp. is considered one of the largest genera of the Mentheae tribe as is the *Salvia* genus, and grows in Southern Europe and in central Asia [38]. Commonly known as “catnip” or “catmint”, these species are traditionally used in human medicine to treat many disorders especially due to the presence of nepetalactone. Moreover, *Nepeta* spp. are used for ornamental and, sometimes, culinary purposes [39,40]. *Nepeta* × *faassenii* (N × faas.) is a garden plant produced by crossbreeding of two Mediterranean species: *N. mussinii* Spreng. Ex henckel and *N. nepetella* L. The EOs of *Nepeta* species are used in food, medicine and perfume industries, and the one obtained from N × faas aerial parts is characterized by two nepetalactones and 1,8 cineole [41]. The last studied species was *Monarda didyma* (M. did), that belongs to the genus which encompass 18 species, endemic to North America [42]. The economic relevance of these plants is related not only to the presence of the EO rich in active compounds [42], but also to the use of leaves as a flavouring agent in the food industry [43].

**Figure 1:**
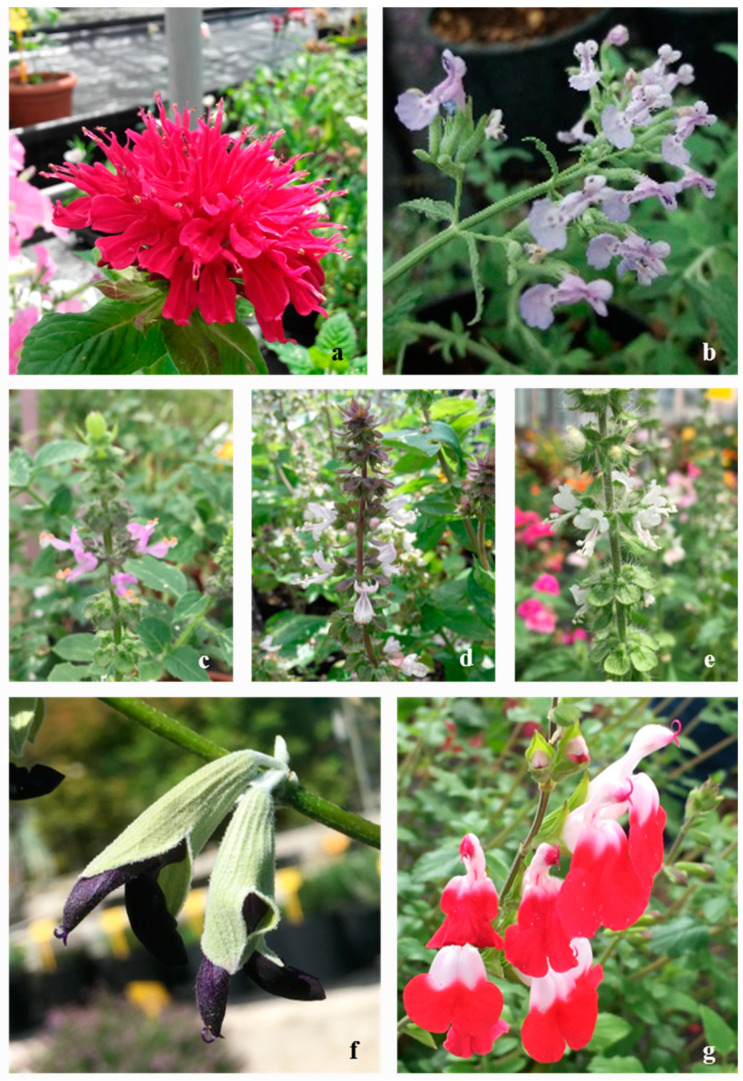
Selected flowers belonging to Lamiaceae family: (**a**) *Monarda didyma* ‘Fireball’ (M. did), (**b**) *Nepeta* × *faassenii* “Six Hills Giant” (N. × faas), (**c**) *Ocimum basilicum* ‘Blue Spice’ (Ob-BS), (**d**) *O. basilicum* ‘Cinnamon’ (Ob-Cn), (**e**) *Ocimum* × *citriodorum* (Ob-Ct), (**f**) *Salvia discolor* (S. disc), (**g**) *Salvia microphylla* ‘Hot Lips’ (S. micro).

The European cross-border cooperation programme between France and Italy INTERREG ALCOTRA “ANTEA” project (N° 1139) was focused on the exploitation of the edible flowers use as functional food and aimed to increase the number of the species used for this purpose. In this study, different species of Lamiaceae family have been considered: *Monarda didyma* ‘Fireball’, *Nepeta* × *faassenii* ‘Six Hills Giant’, *Ocimum basilicum* ‘Blue Spice’, *Ocimum basilicum* ‘Cinnamon’, *Ocimum* × *citriodorum*, *Salvia discolor*, *Salvia microphylla* ‘Hot Lips’ (Figure 1). The selection was based on the flowers’ ornamental value, ease in potting growth, prolonged flowering, and flowers’ aroma and taste (Table 1).

## 2. Results

### 2.1. Bioactive Compounds

Table 2 reported the contents of total crude proteins and soluble sugars (glucose, fructose, and sucrose) in the different flowers. Sugars are an important component of flowers, since the flavor is often related to that content. The Mentheae tribe members resulted in higher sugars content than Ocimeae ones. The two sage species, *Salvia microphylla* Kunth (S. micro) and *Salvia discolor* Kunth (S. disc), characterized by a fruity taste, showed the highest content of sucrose (7.91 and 9.6 mg/g FW) and of hexoses (glucose and fructose) in comparison to *Monarda didyma* L. (M. did) and *Nepeta* × *faassenii* Bergmans ex Stearn (N. × faas). Within the *Ocimum* flowers, *Ocimum × citriodorum* Vis (Ob-Ct) showed the lowest content of soluble sugars (Table 2).

The total crude proteins were higher in the Ocimeae tribe than in the Mentheae members. The three different *Ocimum* spp. showed a proteins percentage in the range of 9.62–16.16%. In the Mentheae tribe only N. × faas evidenced similar proteins percentage (12.69%), while low content was observed in the sage flowers and M. did (3.19–6.29% and 6.79%, respectively).

The carotenoids and anthocyanins amounts were determined and reported in Table 2. The higher contents of carotenoids were detected in the *Ocimum* genotypes, 51.59 µg/g FW in the “Blue spice” (Ob-BS), 68.33 µg/g FW in the “cinnamon” (Ob-Cn) and the highest amount in Ob-Ct (Thai lemon basil) with 81.86 µg/g FW. Within the Mentheae tribe, S. disc had the highest content of carotenoids (61.34 µg/g FW), due to the dark color, while the lowest amount was detected in M. did (1.91 µg/g FW). In relation to the color of flowers, S. disc and M. did showed the highest content of anthocyanins, while the flowers with pale color had lower content, especially Ob-Ct and the Ob-Cn measured 0.03 and 0.06 mg/g FW, respectively. Anthocyanins were abundant in the following sequence: M. did *=* S. disc *>* S. micro *> Ocimum* species (0.98, 0.2, 0.16, 0.06 and 0.03 mg/g FW). The higher polyphenols content was detected in the *Ocimum* species, in the range between 7.42–8.06 mg/g FW, and the lowest amount in the S. micro (Andean sage, 2.41 mg/g FW). The ascorbic acid content (ASA_TOT_, vitamin C), an important nutritional value, was of highest measured in S. micro (2.57 mg/g FW), M. did and N. × faas (2.42 and 2.34 mg/g FW respectively. Lower amounts of total ASA were detected in the S. disc and in the flowers of Ocimeae tribe.

**Figure 2:**
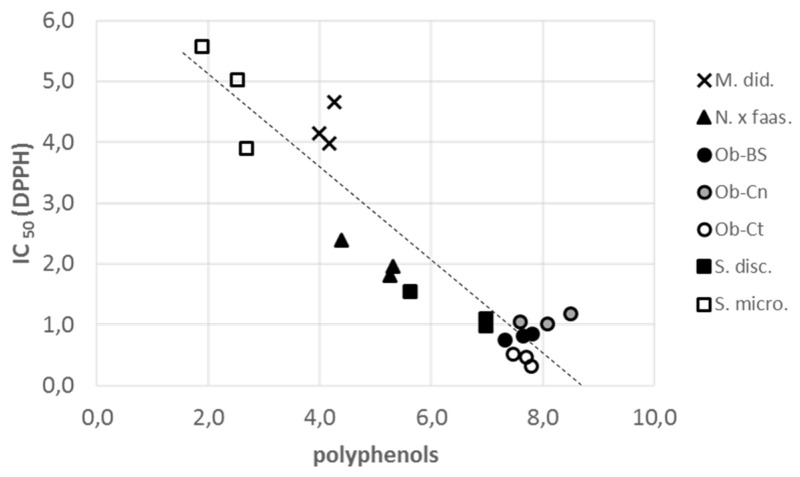
Correlation between polyphenols content in Lamiaceae flowers and the radical scavenger activity (DPPH). Straight line equation: y = −0.7654x + 6.6163; R^2^ = 0.8698.

The radical scavenger activity by DPPH assay was monitored as the IC_50_ value: the highest activity was revealed in Ob-Ct (Thai lemon basil, 0.43 mg/mL), followed by the other two *Ocimum* and the Andean sage (S. disc). The lower antioxidant activity was measured in M. did and S. micro plants. Related to the higher antioxidant activity observed in the *Ocimum* flowers a negative correlation was observed with the highest content of total polyphenols, and is underlined in Figure 2.

### 2.2. Phytochemical Analyses

Overall, 118 chemical constituents were identified in the volatiles from Lamiaceae spp. samples (Table 3) with the number of peaks detected varying between 21 (N. × faas and S. micro) and 51 (*O. basilicum* ‘Cinnamon’, ’Ob-Cn). Sesquiterpene hydrocarbons represented the main class in all *O. basilicum* varieties as well as in S. disc (58.0% in S. disc to 77.9% in Ob-Ct), nevertheless they did not have the same characteristic compounds. β-caryophyllene, which was the only compound in common among all the studied species, represented the highest amount in both S. disc (36.2%) and Ob-Ct (23.7%). Ob-BS evidenced β-bisabolene (26.2%) as main constituent, while germacrene D (17.3%) and β-elemene (16.8%) prevailed in Ob-Cn. The presence of these latter constituents is conspicuous in all the previous species even though with different amounts, except for β-bisabolene, which was almost the exclusive compound of Ob-BS, present with lesser amount in S. disc (4.0%).

More than the half of M. did volatile organic compounds (VOCs) was represented by oxygenated monoterpenes (57.3%), especially constituted by thymol (19.4%) and its methyl ether (19.9%) together with linalool (17.1%). This plant species showed also a good amount of monoterpene hydrocarbons (29.0%), with both *o*-cymene and γ-terpinene as the same highest amount (13.3%).

N. × faas aroma profile was divided into two classes of compounds: oxygenated monoterpenes (OM), which was the predominant one (66.8%), and sesquiterpene hydrocarbons (SH, 31.3%). This species showed β-caryophyllene (19.0%) as the most abundant sesquiterpene together with germacrene D (8.0%). Furthermore, *cis*-*trans*-nepetalactone, an iridoid monoterpenoid (64.2%), was the chief constituent seen that it represents more than 96% of OM class.

The second species of the *Salvia* genus (*S. microphylla*) showed a heterogeneous profile because all the classes were present. In fact, the EO composition evidenced the presence of MH (36.8%), OM (25.6%), OS (21.2%) and SH (14.7%) in this decreasing order. This species was characterized by limonene (25.8%) followed by isobornyl acetate (14.3%) and guaiol (11.5%) as principal components.

### 2.3. Multivariate Explorer Analyses

**Figure 3:**
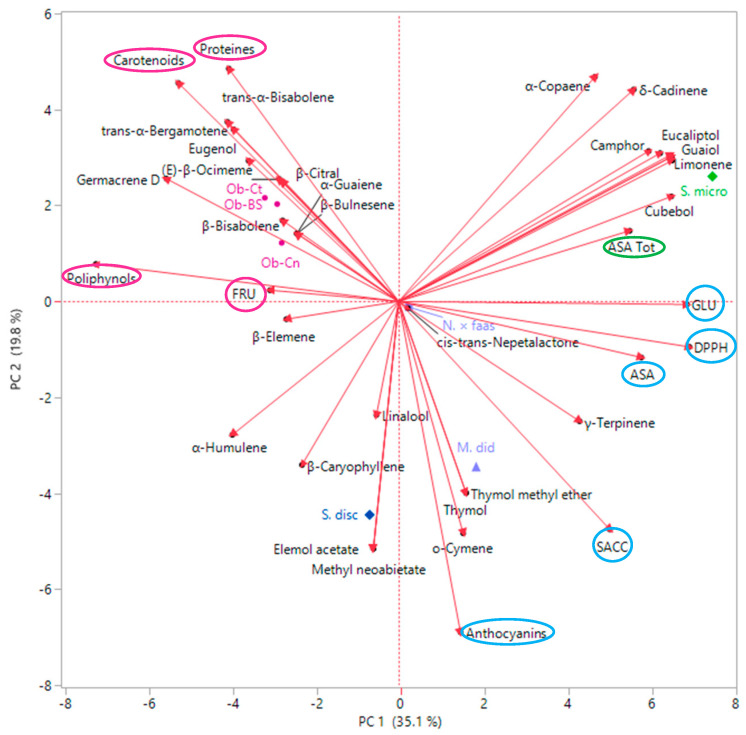
Principal component analysis (PCA) plot depicting phytochemical proximities among VOCs of the studied spp.

Principal component analysis (PCA) was performed with the spontaneous emission compounds present in a percentage greater than 3% in addition to the nutritional values of flowers. The result of this multivariate analysis (Figure 3) where the first two axes account for 54.9% for a correlation matrix, showed two first macro groups, one with positive loading on PC1 and the other one with a negative loading in the same axis. All *Ocimum* varieties, scored negatively along PC1, were located in the upper left quadrant. This loading was generated mostly by the content of both distinguished compounds such β-citral in Ob-Ct (5.5%), (*E*)-β-ocimene (19.8%), eugenol (6.9%) and β-bisabonene (26.2%) in Ob-BS, and common compounds such as *trans*-α-bergamotene (11.6% and 6.4%, respectively), *trans*-α-bisabolene (15.7% and 17.3%, respectively), in addition to their nutritional value as regards proteins (13.8 in Ob-Ct and 16.16 in Ob-BS, respectively) and carotenoids (81.86 in Ob-Ct and 51.59 in Ob-BS respectively). Ob-Cn, even though it had a negative loading along PC1 and plotted in the same quadrant as the other two varieties, was slightly separated from them. In fact, this basil was distinguished by the presence of α-bulnesene (9.5%) and α-guaiene (9.0%) together with the high value of polyphenols (8.06 mg/g FW) and fructose (FRU) (6.85 mg/g FW). S. disc, with its negative loading along both axes, was positioned deep down in the left quadrant by dint of characteristic compounds: elemol acetate (9.0%) and methyl neoabietate (6.3%), together with their amount in saccharose (SACC) and anthocyanins. In the opposite quadrant relative to the Y-axis two out of three remaining species were present: M. did and N. × faas. These species were scored positively along PC1 and negatively along PC2. This position is mainly due to the main compounds as for *Monarda* and *Nepeta*. S. micro was the only sample with a positive loading on both PC1 and PC2, and it was located in the upper right quadrant because both its main constituents were previously cited as well as other specific compounds such as camphor (6.5%), α-copaene (6.3%), δ-cadinene (5.3%), eucalyptol (4.8%) and cubebol (3.0%), with the addition of glucose (GLU) and ASA_TOT_ content.

**Figure 4:**
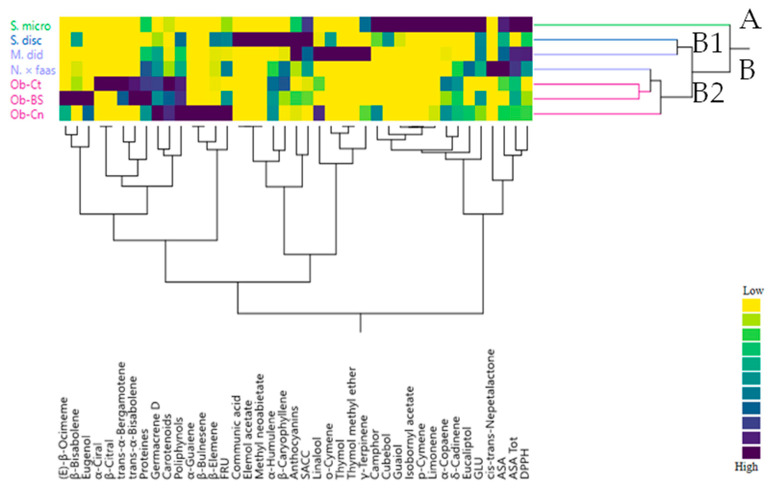
Dendrogram of cluster hierarchical analysis performed on VOCs from the studied Lamiaceae species.

These results were confirmed by the heat map of the two-way HCA analysis (Figure 4) which differentiated *S. microphylla* (A) from other studied species gathered together in the group (B). This latter was further divided into two clusters. The cluster B1 was composed by S. disc and M. did, which in spite they showed a VOC with different compositions, the two species pointed out the highest amount in anthocyanins (0.98 in both spices). The cluster B2 included the remaining plant species. Including *Nepeta* with basil varieties was not strange, owing to fact that this species shared with basil its high percentage of germacrene D as well as proteins and carotenoids.

### 2.4. Essential Oil (EO) Analysis

The different constituents of the EOs from the seven Lamiaceae species studied herein, identified by gas chromatography-mass spectrometry (GC-MS) analysis, are reported in Table 4. Ninety-five compounds were present accounting for 92.7% in *Nepeta* to 100% of the total identification in the oil composition of Ob-BS. The striking thing was the drastic decrease of the number of identified peaks in all the *Ocimum* varieties. This decrease was about 58% in Ob-BS to more than 85% in Ob-Cn.

Another important thing to note was how the fragile and thermosensitive constituents decomposed into artefacts due to the heating during the hydrodistillation. All the *O. basilicum* volatiles were dominated by sesquiterpene compounds which were biosynthesized by the mevalonic acid (MVA) pathway, while the EO distillation originated the volatile monoterpenes (C10) [44]. This is because the two varieties of basil, Ob-Cn and Ob-Ct, showed OM as the main class of compounds in their EOs (72.3% and 52.4%, respectively) except for the Ob-BS that seemed not to be affected by heating since the EO profile evidenced SH (77.4%) as in VOCs. Linalool (48.6%) and terpinene-4-ol (23.7%) were the main monoterpenes in Ob-Cn; α- and β-citral in Ob-Ct (32.2% and 18.8%, respectively). This latter species evidenced also a good percentage of SH (32.6%), represented by *trans*-α-bisabolene (29.3%). This compound (38.7%), together with β-bisabolene (34.4%), were peculiar in Ob-BS.

*M. didyma* showed a trend not very different from its spontaneous emission because it conserved the predominance of the same class of compounds: OM (84.5%). Thymol (68.6%) became the chief compound, while thymol methyl ether completely disappears. By contrast, *Nepeta* evidenced aliphatic hydrocarbons as the most abundant class (NT, 41.2%) together with a good amount of OS (35.2%). In detail of composition, caryophyllene oxide (17.2%) and tetracosane (14.7%) were most abundant constituents.

S. disc had a radically different profile, and its EO was distinguished by its high rate of NTs (77.5%). More than the 63% of this fraction was represented only by three compounds: tetracosane (24.3%), pentacosane (14.6%) and docosyl-isopropyl ether (10.2%). Important was also the amount of apocarotenoids exclusively represented by hexahydrofarnesyl acetone (15.7%). This constituent was also present in the second species of the *Salvia* genus in a notable amount (11.9%). The membership class of this compound was one of the main class in S. micro, even though it was not the prevalent one. In fact, OS (46.1%) and OM (27.0%) were mainstream. Davana ether (16.3%) and carvacrol (10.9%) showed to be the most representative compounds.

### 2.5. Multivariate Explorer Analyses

**Figure 5:**
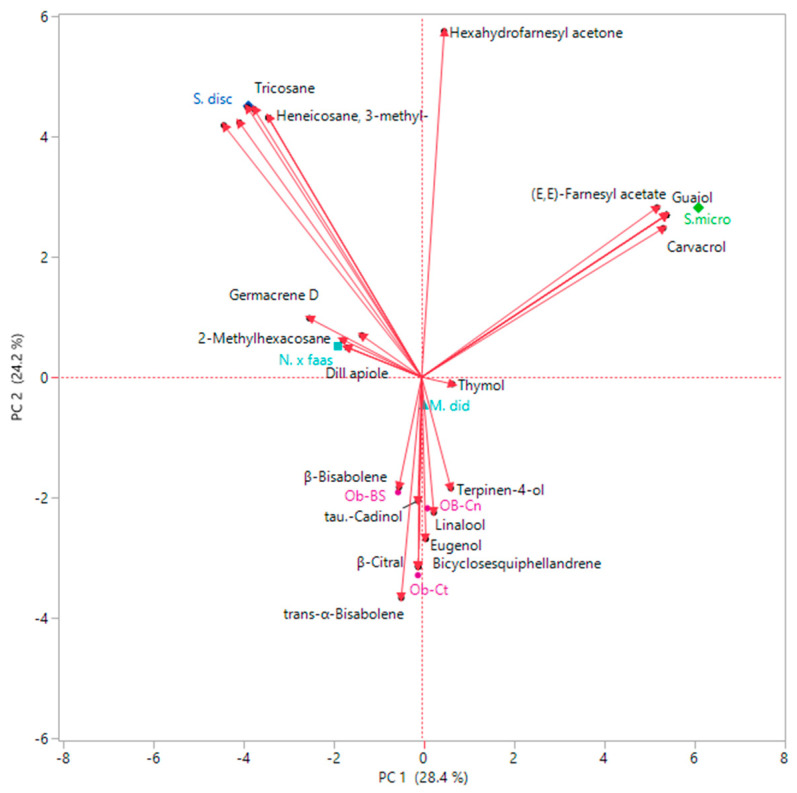
PCA plot depicting phytochemical proximities among the essential oils (EOs) of the studied spp.

The PCA analysis performed with compounds of EOs > 3% was reported in Figure 5. The first two axes account for more than the half (52.6%) of a correlation matrix. Here PC2 plays a key role in the agglomeration of the species rather than PC1. In fact, two macro groups were present: S. micro, S. disc and N × faas were of positive loading on PC2 while the remaining ones were of negative loading. It is interesting to note that only S. micro was positioned in the upper right quadrant (load positively in both axes) and this was due to the exclusive compound (*E,E*)-farnesyl acetone and guaiol as well as its high amount of carvacrol. The species with the highest percentage of NTs, S. disc and N. × faas, were positioned on the opposite quadrant. All the basil species together with *Monarda* were located in the borderline along Y-axis, except for Ob-BS which shifted a little to the left, this was because of its content in β-bisabonene.

**Figure 6:**
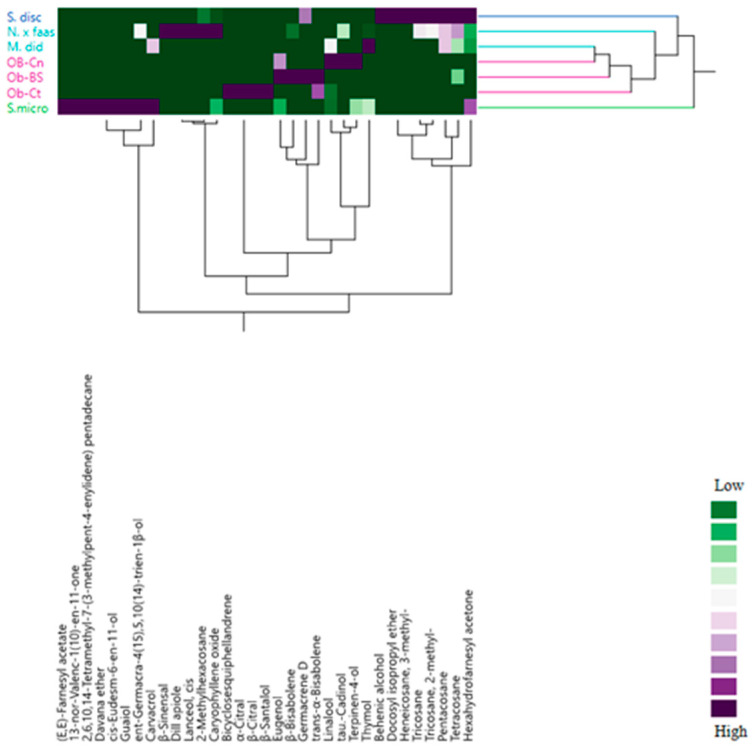
Dendrogram of cluster hierarchical analysis performed on EOs from the studied Lamiaceae species.

The heat map of the two-way HCA analysis (Figure 6) confirmed what observed in PCA analysis and distinguish S. micro from the others (group I). The second group II was further divided into two subgroups: II.1 homogeneous constituted only by S. disc; II.2 which gathered M. did with all the basil species.

## 3. Discussion

### 3.1. Bioactive Compounds

Carbohydrates are the most abundant macronutrient in edible flowers, reaching even 90% of *Rosa micrantha*’s dry weight [13,45]. Nectar is a relevant source of soluble sugars [14], and it is composed of water, sucrose, glucose, fructose, and traces of 10 minor sugars [46]. Most Lamiaceae flowers are known to produce nectar in significant amounts and several species are cultivated as melliferous plants [47,48]. In our study, *Salvia* spp. and M. did flowers contained the highest quantities of glucose and sucrose, while Ob-Cn was characterised by the highest amount of fructose (Table 2). Soluble sugars were poorly represented in Ob-Ct, compared to the other six flowers under evaluation (Table 2). Sucrose amount in M. did was fully comparable with the results obtained by Stefaniak and Grzeszczuk [49] who analysed the same species. However, some discrepancy in the amount of total reducing sugars was evidenced. This could be due to other components of reducing sugars and/or to the genetic difference between M. did and plant material, the origin of the outset plant material and the cultivation methods used in other reports. Very few studies were performed on the detection of soluble sugars in *O. basilicum* edible flowers. Shanaida et al. [50] quantified total soluble sugars and reducing sugars in *O. americanum*, with similar range of contents as those presented here.

Usually, in edible flowers carbohydrates are followed by proteins, ranging between 2.0 and 52.3 g/100 g DW (reviewed in [13]). In our work, these primary metabolites were of the highest amount in *Ocimum* spp. and N. × faas, exceeding 10% of the flowers’ dry weight. Similar results were obtained analysing other well-known edible flowers, such as *Allium schoenoprasum* [51] and *Cucurbita pepo* [52], although these flowers belong to different families. However, previous work on flowers of *M. didyma* showed higher percentage of proteins, due to the cultivation systems [49].

Secondary metabolites are classified in phenolics, terpenes and steroids, and alkaloids [53]. They are usually involved in the adaptation of plants to their environment, playing a role in plant defense against biotic and abiotic stresses [53], ultraviolet radiation and oxidants [54]. Flowers assigned on these molecules the role to attract pollinators as well as the fragrance and brightness [53,54,55]. Flowers’ colours are determined by flavonoids (mostly anthocyanins), betalains and carotenoids [53,56], that often contribute in mixture to the final flowers’ hue [56]. Carotenoids are involved in yellow, orange and red flowers’ pigmentation [57], while anthocyanins are mainly responsible for the bluish to purple and reddish colors [54]. Betalains are the yellow and violet pigments that replace anthocyanins in plants belonging to the order Caryophyllales [58] and, for this reason, they were not evaluated in this work. In our study, carotenoids were higher in S. disc and *Ocimum* spp. flowers (Table 2) than in the other three varieties under evaluation and some species reported in literature, such as the pale colored *Telosma minor* (Andrews) W. G. Craib and *Piper retrofractum* Vahl (12.9 each μg/g DW) [59], as well as other 11 species (0.020–0.992 μg/g FW), including *Lavandula angustifolia* Mill. and *Salvia spendens* Sellow ex Roem. et Shult. [60].

On the other hand, the examined flowers contained less carotenoids than *Hemerocallis* × *hybrida* Hort., *Mimulus* × *hybridus* ‘Magic Yellow’, and black *Dianthus chinensis* L. ‘Chianti’ [49]. In fact, all of them, with the only exception for *D. chinensis* ‘Chianti’, were decribed as flowers with intense yellow, orange or red colourations. This feature makes these flowers very different from those described herein, which are characterised by softer tones. Anyway, regarding M. did, there was a strong discrepancy between our carotenoid quantification and the one obtained by Stefaniak et al. [49] (1.91 vs. 167.20 μg/g FW). This could be due to different genetic background and cultivation systems.

S. disc and M. did were rich in anthocyanins, as their colour suggested. This class of metabolites is higher in these two flowers than in *Begonia semperflorens* Link and Otto (0.05 mg/g FW), *Fuchsia hybrida* Hort. Ex Siebert and Voss (0.08 mg/g FW) and *Pelargonium peltatum* (L) L’Hér. (0.14 mg/g FW), which are characterised by red petals [61]. Nevertheless, *D. chinensis* ‘Chianti’ and *M. didyma* contain more than 2 mg/g FW of anthocyanins [49]. These last results were obtained with different methods, therefore the comparison may be not similar.

Between the species studied herein, S. disc and *Ocimum* spp. showed the highest content in total polyphenols (TPC). The same metabolites are comparable between S. micro (2.41 mg/g FW) and *S. splendens* (2.16 mg/g FW) [60]. To the best of our knowledge, the TPC of *O. basilicum* flowers was reported in only one paper [62], with the quantification of three different basil cultivars (‘Subja’, ‘Holy green’ and ‘Red rubin’) in freeze-dried samples, making difficult the comparison with the fresh flowers used in this work. No studies were published on the TPC in N. × faas flowers, but some data are available for other species belonging to the same genus, such as *N. cataria* L. [63] and *N. nepetella* L. [64]. Dried flowers of these two species are characterised by 2- and 4-fold more TPC than N. × faas fresh flowers [63,64].

Ascorbic acid (vitamin C, ASA) is known to take part in essential human biochemical and physiological processes. This molecule plays a relevant role in the development and maintenance of connective tissues, in bone formation and wound healing [65]. ASA is also involved in several metabolic pathways, in the proper functioning of the immune system and it protects the human body from free radicals’ damages [65]. However, human organisms are not able to synthetize this vitamin, since humans lack the terminal enzyme of its biosynthetic pathway [66]. For this reason, ASA must be present in a well-balanced diet, and the consumption of edible flowers can help to supply the EU daily requirements intakes (80 mg per days) [67]. In fact, *Tagetes tenuifolia* Cav. and *Viola tricolor* L. are considered good sources of vitamin C, containing 241.20 and 182.16.20 mg/100g FW respectively [60].

In this work, M. did, S. micro and N. × faas were characterised by higher levels of total (ASA_TOT_) and the reduced form (ASA) than the other species under evaluation (Table 2). Nevertheless, the amounts of vitamin C were very low, and due to their small size, thousands of flowers would be needed to reach the EU recommended intakes. However, compared to other Lamiaceae flowers, these two varieties and N. × faas were characterized by ASA_TOT_ content similar to some *Agastache* spp. [17]. On the other hand, M. did and S. micro contained around −18 and −15 fold less vitamin C than *S. splendens* and L. angustifolia [60].

Carotenoids, TPC and ASA are known as antioxidant molecules [68,69]. The radical scavenging activity of the flowers under evaluation was highest in the Ocimeae tribe. This parameter is remarkable in Ob-Ct species, since it is higher than other edible flowers such as *Agastache* ‘Blue Boa’ (IC_50_ 0.86 mg/mL) [17], *Crithmum maritimum* L. (IC_50_ 0.71 mg/mL) [70], and *Centaurea cyanus* L. (IC_50_ 0.79 mg/mL) [71]. A strong correlation between polyphenols and radical scavenging activity (R^2^ = 0.8698) (Figure 2) were observed, as already evinced in other edible flowers, such as *Bellis perennis* L. [72], *Calendula officinalis* L. [73], and 19 Chinese species [74].

### 3.2. Spountaneous Emissions

In this study, we evaluated the aroma profile spontaneously emitted from the seven selected Lamiaceae flowers. The analyses of the volatile organic compounds increased our knowledge concerning their ecological role. In spite of this, the species that have been investigated for their spontaneous emission were too negligible compared to the high number of plants present in nature. The spontaneous emission of *Ocimum basilicum* (Ob) was already widely studied [75,76,77,78,79], but only a few papers investigated its varieties and none of these works reported the varieties studied herein. In 2008, Klimánková et al., [80] evaluated five cultivars of basil green cultivar I (Prava zelena), green cultivar II (Trpaslici), red cultivar III (Cinamonette), red cultivar IV (Purple Opaal), and red cultivar V (Rot), and their volatile composition was characterized by linalool, methyl chavicol, eugenol, bergamotene, and methyl cinnamate. A more recent study reported the SPME components of two basil varieties (Violetto and Genovese) where linalool (18.94% and 22.57% respectively), eugenol (3.95% and 15.02%, respectively) and methyl eugenol (39.17% and 19.39%, respectively) were identified as the main constituents [77]. On the contrary, the whole spontaneous emission of Malaysian *O. basilicum* flower was represented by estragole (88.18%) [78]. In the three *Ocimum* varieties studied herein only linalool and eugenol were detected even though their presence was not detected in all varieties (Ob-Cn and Ob-Ct for linalool and Ob-Cn and Ob-BS for eugenol) obviously with lesser percentage. Bergamoptene was observed in Ob-BS studied here. Methyl eugenol and methyl cinnamate were not present while chavicol (= eustragol) was of lesser amount and only present in Ob-BS.

To the best of our knowledge, no works considered the aroma profile of *M. didyma* or any other species belonging to the same genus.

Concerning the studied *Nepeta* × *faassenii* no work was found related to its VOC composition. Moreover, this genus seemed to be not attractive seeing the scant reports in this context. The first work found dates back to 2010 when the authors used the Proton-Transfer-Reaction Mass Spectrometry (PTR-MS) method to evaluate the VOCs of three *Nepata* species cultivated in vitro. High concentration of nepetalactone was evidenced in *N. sibirica* L. and especially in *N. rtanjensis* Diklic and Milojevic shoot cultures, even though this constituent was detected in traces in *N. nervosa* Royle ex Benth. [81]. Recently Yayali and collaborators [82] investigated the Turkish *Nepeta conferta* Hedge and Lamond and reported that *p*-cymene (25.5%), eucalyptol (9.8%), limonene (5.0%), sabinene (4.8%), carvacrol (3.7%), (*E*)-linalool oxide (3.3%), (*Z*)-linalool oxide (3.0%) [82]. Moreover, Barhoumi et al. [83] studied the VOCs of two wild *Nepeta curviflora* Boiss originating in two Jordan regions (Salt, Northwest of Amman capital and Irbid, in the Northern of Jordan). Fully expanded flowers from Salt were characterized by a high SH content (75.94%) especially represented by *trans*-caryophyllene (26.50%) and OMs (18.53%) represented mainly by 4aα,7α,7aα-nepetalactone (12.74%). The main sesquiterpene hydrocarbons detected in the emission profiles of the flowers from the northern species, collected during the full blossoming stage, included β-bourbonene (19.45%), α-copaene (13.37%) and bicyclogermacrene (7.09%) [83]. All these compounds were completely absent in the VOCs of the species studied herein, except for nepetalactone and trans-caryophyllene.

No work was also found in both the studied species of *Salvia* genus, notwithstanding numerous published works on this subject [84,85,86,87,88,89,90,91,92,93,94]. The two studied species S. disc and S. micro, originating from South America, were grown under uniform conditions in CREA-Sanremo (Italy). As reported by Ascrizzi et al. [93], with the exception of only 3 species, all the South American studied plants were rich in SHs with a percentage ranging from 54.4% and 96.5% and showed β-caryophyllene and germacrene D as the most abundant ones. This is in a total agreement with the class of compounds in S. disc also for the presence of these two compounds because the first one showed a similar amount likewise what reported by the cited work while the second one had a very low amount. The same work underlined the presence of other sesquiterpene constituents in South American species such valencene, α-copaene, cis-muurola-3,5-diene, β-bisabolene and γ-muurolene. Except for β-bisabolene, all the other compounds were lacking. S. micro profile followed the same trend of one of the exceptions of South American species: *S. dorisiana* [93]. In this latter the whole volatile emission profile was mainly composed by MH (77.9%), with limonene of the most abundant compound (11.65%).

### 3.3. Essential Oils

Numerous reports are present in the literature on the EO composition of *O. basilicum* taxa which are very complex and show wide compositional variability according to the presence of several chemotypes within the species and according to the varied climatic/geographical conditions and agronomic practices [95]. Regardless of these factors, monoterpenes were commonly distributed in basil EOs and the linalool percentage was very high (ranging from 29.2% to 75.9%) as in the ‘Cinnamon’ variety, reported by many studies [96,97,98,99,100,101,102,103,104,105,106]. Only few papers reported the EO composition of *O. basilicum* varieties and among these we can find the study of Sajjadi [107], who investigated two Iranian basil varieties (*O. basilicum* L. cv. purple and *O. basilicum* L. cv. Green). Methyl chavicol was the characteristic compounds of both (52.4% and 40.5%, respectively). Although the oil of green basil was characterized by a high content of citral (both neral and geranial, 46.1%), citral was not detected in purple basil oil [107]. The same varieties from Yemen evidenced a completely different behaviour where linalool prevailed in both varieties (44.3% in *O. basilicum* var. *purpurascens* (purple) vs. 46.2% in *O. basilicum* var. *basilicum* (green) [108]. In the same year, another work was published by Tsasi and co-workers [109], where the effect of harvesting was studied in five *O. basilicum* varieties. The Ob-Cn EO were in agreement with *O. basilicum* var. *latifolia* and *O. basilicum* var. *minimum*, cultivated in the field, concerning their linalool content (49.5% and 52.0%, respectively) and with var. *violetto* (11.9) and var. *latifolia* (10.1%) cultivated in the greenhouse regarding the eugenol amount. Among the studied varieties in this work, Ob-Ct was the only one reported by the literature. The first work was done by Turkish scientists, who actually did not study directly this variety, but compared some investigated EOs with a high amount of citral compared to what is found in lemon balm basil, known as *O.* × *citriodorum* or *O. basilicum* var *citriodorum* (a hybrid of *O. basilicum* × *O. americanum*) [110]. Further on, in 2000, another Turkish research team succeeded in the cultivation of *O.* × *citriodorum* and confirmed the domination of neral (43.3%) and geranial (43.4%) in the flower EO [111]. A quite recent work confirmed a good percentage of citral (20.7%) in *O.* × *citriodorum* even though it was not the main compound which was represented on the contrary by nerol (23.0%) [112]. Asian *O.* × *citriodorum* showed the presence of two chemotypes: the first one was rich in geranial/neral, which is the same as this study, and another one with methyl chavicol [113]. The behaviour of the investigated Ob-BS followed the *O. basilicum* var. ‘Blue Spice’ [29].

The studied species of *M. didyma* showed an EO almost exclusively formed by thymol. The richness in this compound was confirmed by many scientific publications. In fact, Fraternale et al. [114], showed the prevalence of thymol (51.7%) and γ-terpinene (14.3%) in the flower EO of *M. didyma*. Also, a *Monarda* species grown in Canada underlined thymol (41.17%), γ-terpinene (15.88%), carvacrol (15.20%), and *p*-myrcene (12.58%) as main constituents [115]. Two other studies published in 2017 reported the EO composition from this plant species cultivated in central Italy: the EO from the flowering aerial parts pointed to thymol (59.3%) and *p*-cymene (10.3%) as major compounds [116], while the second work evidenced thymol 62% [43]. Other *Monarda* species, always cultivated in Italy, were very rich in monoterpenes, but with *o*-cymene (13.42), γ-terpinene (22.15), and carvacrol (13.80%) as the main constituents, and thymol with a lesser amount (5.87%) [42]. The chemical characterisation of the EO from *Nepeta* can be traced back to 1967 when Regnier [117] studied three species and each one showed a different main compound: nepetalactone in *N. cataria* L. (77%), epi nepetalactone in *N. mussini* Spreng. Ex Henckel (70%) and citronellol in *N. citriodora* Dumort. Since then several species were studied. The bulk of investigated plants were distinguished by the presence of a good amount of at least one of the nepetalactone isomers (ranging between 16% to 72%) such as *N. cataria* [118], *N. rtanjensis* [119], *N. cataria* var. *citriodora* and *N. nuda* L. [120]. All these works disagree with what was found in the analysed *Nepeta* × *faassenii* where these compounds were completely absent. The presence of non-terpene compounds was observed in the Lebanese *Nepeta* species such as *N. cilicica* Boiss. ex Benth [121], *N. nuda* ssp. *Pubescens* and *N. curviflora* Boiss [122]. These results did not agree with the data found herein since, despite this class was the main one, the constituents were completely different. Caryophyllene oxide, one of the most important compounds in our *Nepeta* × *faassenii* (17.2%), was evidenced in the higher amount in *N. melissifolia* Lam. and *N. sibirica* (22.06 and 20.35%, respectively) [120]. The only work which analysed the studied *Nepeta* hybrid was that of Ali and his co-workers [123], who found an EO rich in 1,8-cineole. This compound was present in very fewer amount in our study.

Leafing through the literature, the chemical composition of *S. microphylla* EO dates back to 1992 when Chialva et al. [124] identified compounds such as α-pinene, β-pinene, camphene, δ-3-carene, limonene, 1,8-cineole, camphor, borneol, bornyl acetate, (*E*)-caryophyllene, α-copaene, globulol, spatulenol, α-eudesmol and β-eudesmol. Later, Aydogmus et al. [125] observed the presence of β-eudesmol and 8-α-hydroxy-β-eudesmol. In the last decade, two works analysed the EO composition of this *Salvia* spp. The former found that (*E*)-caryophyllene (15.35%), α-eudesmol (14.06%), β-eudesmol (8.74%) and γ-eudesmol (7.64%) were the principal compounds [126], while the latest one evidenced α-eudesmol (20.5%), β-caryophyllene (13.7%) γ-eudesmol (8.2%), spathulenol (7%), and bornyl acetate (6.8%) [127]. In 2019, Wróblewska and collaborators [128], found linalool (46.91%), thymol (17.72%), its methyl ether (6.4%) and *p*-cymene (9.66%). In the current study, the EO composition greatly differed from the others seen before. As far as we know, the *S. discolor* EO profile was reported only in the paper of Sharopov et al. [129] who investigated the German species and underlined its richness in intermediol (57.37%) and (*E*)-caryophyllene (17.81%).

## 4. Materials and Methods

### 4.1. Plant Material and Cultivation

*Monarda didyma* “Fireball” and *Nepeta* × *faassenii* “Six Hills Giant” plants were bought at L’Erbaio della Gorra (Str. Gianardo, 11 Casalborgone, To, Italy,) plant nursery, and then grown in open field for two years in private garden. Cutting were used for plant propagation in greenhouse. Seeds of *Ocimum basilicum* ‘Blue Spice’, *Ocimum basilicum* ‘Cinnamon’ and *Ocimum* × *citriodorum* were provided to the Conservatoire National des Plantes à Parfum, Medicinales et Aromatiques (Milly-la-Forêt, France). *Salvia discolor* and *S. microphylla* “Hot Lips” are currently part of the plants collection at CREA—Research Centre for Vegetable and Ornamental Crops (CREA, Sanremo, IM, Italy, GPS: 43.816887, 7.758900) where they were propagated by cuttings. All the plants used in this work, both deriving from seed or cutting, were cultivated in pots kept in an unheated greenhouse covered with an anti-insect net at CREA, as reported by Najar et al. [17]. Briefly, the plants were cultured in substrate (Hochmoor—Terflor, Capriolo, BS, Italy) with slow release fertilizer (Nitrophoska, Eurochem Agro, Cesano Maderno, MB, Italy) and irrigated with nutrient solution (Ferti 3, Planta-Dȕngemittel, Regenstauf, Germany) every week. Supplemental irrigations with water were carried out according to the needs of the plants and the season in order to avoid water stress to the plants. The plants were grown applying the organic cultivation method (without pesticides), using antagonist insects (Koppert Italia Srl., Bussolengo, VR, Italy) and microorganisms [17]. Full-bloom flowers were picked during their flowering time (see Table 1).

### 4.2. Biochemical Analyses

Fresh flowers were picked early in the morning, divided into three homogeneous biological replica, and stored at −80 °C. Frozen samples (200 mg) were used to quantify total carotenoid [130], total polyphenolic content (TPC) (Folin-Ciocalteu method, according to [17]), and total anthocyanins content [17]. Radical scavenging activity was determined by DPPH assay [131], reporting the results as IC_50_ (mg/mL). Soluble sugars (D-glucose, D-fructose and sucrose), total ascorbate (ASA_TOT_) and reduced ascorbate (ASA) were quantified as described in Najar et al. [17]. All measurements were performed with an ultraviolet (UV)-1800 spectrophotometer (Shimadzu Corp., Kyoto, Japan). Total nitrogen content determination was performed by Kjeldhal method following the protocol described in Jones et al., [132]. Data were reported as percentage of crude protein content, obtained by multiplying the percentage of nitrogen by 6.25 as conversion factor (%N × 6.25).

### 4.3. Phytochemical Analysis

A fresh flower of each plant was picked (an average of 0.5 to 1 g), placed separately in a glass conical flask (20 mL) and sealed with a cap provided with aluminium foil for 30 min (equilibration time). The evaluation of VOC emission was performed with the use of 100 μm polydimethylsiloxanes (PDMS) fibre manufactured by Supelco Ltd (St. Louis, MO, USA). Prior to the analyses, the fibre was conditioned according to the manufacturer’s instruction, at 250 °C for a duration of 30 min in the injector of a gas chromatograph. Exposition of the fibre in the headspace phase of the samples took place for 15 min at a temperature of 23 °C. Subsequently the fibre was reinserted back into the needle and immediately transferred to the injector of the gas chromatograph (temperature 250 °C), where the analytes were thermally desorbed for a duration of 30 min. The composition of the compounds desorbed from SPME fibre was examined using GC-MS.

Essential oil (EO) was extracted from fresh flowers even though the weight of these plant material was barely sufficient to undertake a microdistillation. Therefore, the fresh flowers were separately hydrodistilled for 2 h using a micro-Clevenger like apparatus as recommended by the European Pharmacopeia [133]. The yield of the EOs were very low and were collected directly in high-performance liquid chromatography (HPLC)-grade *n*-hexane and immediately analysed by GC-MS.

GC-MS analyses were performed with an Agilent 7890B gas chromatograph (Agilent Technologies Inc., Santa Clara, CA, USA) equipped with an Agilent HP-5MS (Agilent Technologies Inc., Santa Clara, CA, USA) capillary column (30 m × 0.25 mm; coating thickness 0.25 μm) and an Agilent 5977B single quadrupole mass detector (Agilent Technologies Inc., Santa Clara, CA, USA). Analytical conditions were as follows: injector and transfer line temperatures 220 and 240 °C, respectively; oven temperature programmed to raise from 60 to 240 °C at 3 °C/min; carrier gas helium at 1 mL/min; injection of 1 μL (0.5% HPLC grade *n*-hexane solution); split ratio 1:25. The acquisition parameters were as follows: full scan; scan range: 30-300 m/z; scan time: 1.0 sec. Identification of the constituents was based on a comparison of the retention times with those of the authentic samples, comparing their linear retention indices relative to the series of *n*-hydrocarbons. Computer matching was also used against commercial [134,135] and laboratory-developed mass spectra library built up from pure substances and components of known oils and MS literature data [135,136,137,138,139,140].

### 4.4. Statistical Analysis

Biochemical results were statistically analysed using either Tukey’s honest significant difference (HDS) or the Games-Howell test according to the homogeneity of variance (Levene’s test) [141]. The analyses were performed using IBM SPSS software (IBM Corp. Released 2017. IBM SPSS Statistics for Windows, Version 25.0. Armonk, NY: IBM Corp).

Linear correlation between polyphenols and radical scavenging activity were determined using Microsoft Excel ^®^ 2013 (Microsoft Corporation, Redmond, WA, USA).

Multivariate explorer, principal component and hierarchical clustering analyses were carried out which allowed the co-evaluation of all variables [142]. For each treatment, the chemical compounds and their proportions (%) were plotted in Excel spreadsheets. Compounds present with amounts less than 5% were omitted from the analysis. The data were transformed by orthogonal rotation into latent variables named as the principal components. These are linear combinations of original variables created from the eigenvalues of the data correlation matrix. The Euclidean distance was used as a dissimilarity metric to represent the straight-line distance between the centroids of each cluster of chemical compounds identified in EO analysis. The unweighted pair group method with arithmetic averages (UPGMA) was used to cluster the compounds. The results were presented in a dendrogram that characterized the clusters. Both analyses were run in the JMP software package 13.0.0 (SAS Institute, Cary, NC, USA).

## Figures and Tables

**Table 1:** Main botanical information of the examined Lamiaceae flowers. * = The taste was evaluated by CREA (Research Centre for Vegetable and Ornamental Crops, Sanremo, IT) and CREAM (Chambre d’Agriculture des Alpes-Maritimes, Nice, FR), as one of the ANTEA project goals.

Acronyms	Species/Hybrid	Variety/Genotype	English Name	Flowering Period	Taste *
M. did	*Monarda didyma* L.	Fireball	Bee balm	Jun-Aug	Sweet oregano
N. × faas	*Nepeta* × *faassenii* Bergmans ex Stearn	Six Hills Giant	Catmint	Mar-Nov	Strong aromatic
Ob-BS	*Ocimum basilicum* L.	Blue Spice	-	Apr-Nov	Spice
Ob-Cn	*Ocimum basilicum* L.	Cinnamon	Cinnamon basil	Apr-Nov	Cinnamon
Ob-Ct	*Ocimum* × *citriodorum* Vis	-	Thai lemon basil	Apr-Nov	Lemon peel
S. disc	*Salvia discolor* Kunth	-	Andean sage	Jan-Nov	Black currant and pine nut
S. micro	*Salvia microphylla * Kunth	Hot Lips	-	Feb-Oct	Floral and fruity

**Table 2:** Determination of primary and secondary metabolites in the seven studied flowers of Lamiaceae family. Data are presented as means ± standard error (SE, n = 3). Abbreviations: FW = fresh weight; DW = dry weight; GAE—gallic acid equivalents; CE—± catechin equivalents; ME—malvin equivalents; sig.= significant post hoc test at *p* < 0.05.

Parameters	*Monarda didyma* ‘Fireball’ (1)	*Nepeta × faassenii* ‘Six Hills Giant (2)	*Ocimum basilicum* ‘Blue Spice (3)	*Ocimum basilicum* ‘Cinnamon’ (4)	*Ocimum × citriodorum* (5)	*Salvia discolor* (6)	Salvia microphylla ‘Hot Lips’ (7)	Sig.
Primary metabolites								
D-Glucose (GLU) mg/g FW	5.07 ± 0.16	4.36 ± 0.40	4.70 ± 0.35	3.49 ± 0.12	3.03 ± 0.11	5.02 ± 0.19	7.60 ± 0.50	1*vs*4,5,7//2*vs*7//3*vs*5,7// 4*vs*1,6,7//5*vs*1,3,6,7 6*vs*4,5,7//7*vs*1,2,3,4,5,6
D-Fructose (FRU) mg/g FW	2.19 ± 0.22	4.11 ± 0.46	3.58 ± 0.15	6.85 ± 0.64	2.10 ± 0.08	3.96 ± 0.21	2.46 ± 0.27	1*vs*2,4,6//2*vs*1,4,5//3*vs*4// 4*vs*1,2,3,5,6,7//5*vs*2,4,6// 6*vs*1,4,5//7*vs*4
Sucrose (SUC) mg/g FW	6.66 ± 0.56	4.46 ± 0.02	2.44 ± 0.29	1.27 ± 0.11	1.60 ± 0.05	9.60 ± 0.84	7.91 ± 0.43	1*vs*2,3,4,5,6//2*vs*1,4,5,6,7// 3*vs*1,6,7//4*vs*1,2,6,7//5*vs*1,2,6,7// 6*vs*1,2,3,4,5//7*vs*2,3,4,5
Crude protein (% DW)	6.79 ± 0.16	12.69 ± 0.25	16.16 ± 0.16	9.62 ± 0.12	13.81 ± 0.00	3.19 ± 0.31	6.29 ± 0.16	1*vs*2,3,4,5,6//2*vs*1,3,4,5,6,7// 3*vs*1,2,4,5,6,7//4*vs*1,2,3,5,6,7// 5*vs*1,2,3,4,6,7//6*vs*1,2,3,4,5,7// 7*vs*2,3,4,5,6
Secondary metabolites								
Total carotenoids (TCar) μg/ g FW	1.91 ± 0.02	6.92 ± 0.98	51.59 ± 6.48	68.33 ± 3.10	81.86 ± 1.48	61.34 ± 0.09	4.25 ± 0.53	1*vs*3,4,5,6//2*vs*3,4,5,6// 3*vs*1,2,4,5,7 4*vs*1,2,3,7//5*vs*1,2,3,6,7 6*vs*1,2,5,7//7*vs*3,4,5,6
Total anthocyanins (TAnth) mg ME/g FW	0.98 ± 0.04	0.09 ± 0.00	0.16 ± 0.00	0.06 ± 0.00	0.03 ± 0.00	0.98 ± 0.08	0.20 ± 0.02	1*vs*2,3,4,5,7//2*vs*1,3,4,5,6,7 3*vs*1,2,4,5,6//4*vs*1,2,3,6,7 5*vs*1,2,3,6,7//6*vs*2,3,4,5,7 7*vs*1,2,4,5,6
Total polyphenols (TPC) mg GAE/g FW	4.14 ± 0.08	5.11 ± 0.21	7.42 ± 0.13	8.06 ± 0.18	7.63 ± 0.14	6.53 ± 0.29	2.41 ± 0.18	1*vs*3,4,5,6,7//2*vs*3,4,5,6,7 3*vs*1,2,7//4*vs*1,2,6,7//5*vs*1,2,7 6*vs*1,2,4,7//7*vs*1,2,3,4,5,6
Ascorbic acid reduced form (ASA) mg AsA/100 g FW	1.36 ± 0.07	1.77 ± 0.05	0.56 ± 0.03	0.81 ± 0.05	0.77 ± 0.10	0.99 ± 0.05	1.64 ± 0.05	1*vs*2,3,4,5,6//2*vs*1,3,4,5,6 3*vs*1,2,6,7//4*vs*1,2,7//5*vs*1,2,7 6*vs*1,2,3,7//7*vs*3,4,5,6
Total ascorbic acid (AsA_TOT_) mg AsA_TOT_ /100 g FW	2.42 ± 0.03	2.34 ± 0.44	1.76 ± 0.07	1.45 ± 0.21	1.61 ± 0.05	1.14 ± 0.07	2.57 ± 0.31	1*vs*4,5,6//2*vs*4,6//3*vs*7 4*vs*1,2,7//5*vs*1,7 6*vs*1,2,7//7*vs*3,4,5,6
Radical scavenging assay (IC_50_ DPPH-mg/mL)	4.26 ± 0.20	2.05 ± 0.17	0.81 ± 0.03	1.08 ± 0.05	0.43 ± 0.05	1.20 ± 0.17	4.83 ± 0.49	1*vs*2,3,4,5,6//2*vs*1,3,4,5,7 3*vs*1,2,7//4*vs*1,2,7//5*vs*1,2,7 6*vs*1,7//7*vs*2,3,4,5,6

**Table 3:** Volatile chemical composition (by headspace solid phase microextraction, HS-SPME) of flowers from the studied Lamiaceae species (n = 3) ^1^.

	Compounds	Class	RI (esp)	RI (lit)	M. did.	N. × faas	Ob-BS	Ob-Cn	Ob-Ct	S. disco	S. micro
					Relative Abundance %						
1	ethyl *iso*valerate	NT	854	856 $	-	-	0.5 ± 0.35 *	-	-	-	-
2	β-myrcene	MH	991	988	0.8 ± 0.23	-	-	0.1 ± 0.07	-	-	-
3	oxime, methoxy phenyl	NT-N	926	-	-	-	-	-	-	1.1 ± 0.55	-
*4*	α-thujene	MH	929	924	-	-	-	0.1 ± 0.10	-	-	-
*5*	α-pinene	MH	937	932	-	-	-	0.1 ± 0.10	-	-	0.4 ± 0.40
6	camphene	MH	952	946	-	-	-	0.1 ± 0.08	-	-	-
7	β-thujene	MH	966	971 $	-	-	-	-	-	0.4 ± 0.37	-
8	β-myrcene	MH	991	988	-	-	-	0.1 ± 0.10	-	-	-
9	α-phellandrene	MH	1005	1002	-	-	-	0.1 ± 0.06	-	-	-
10	(+)-4-carene	MH	1009	1004 $	1.6 ± 0.19	-	-	-	-	-	-
11	(E,E)-2,4-nonadiene	NT	1014	1014 $	-	-	-	0.1 ± 0.08	-	-	-
*12*	α-terpinene	MH	1017	1014	-	-	-	0.1 ± 0.10	-	-	-
13	*o*-cymene	MH	1022	1022	13.3 ± 3.98	-	-	-	-	2.0 ± 0.96	-
14	*p*-cymene	MH	1025	1020	-	-	-	-	-	-	4.0 ± 0.23
15	limonene	MH	1030	1224	-	-	-	0.5 ± 0.09	-	-	25.8 ± 2.11
16	eucaliptol	OM	1032	1026	-	2.3 ± 0.17	-	0.6 ± 0.06	-	-	4.8 ± 0.10
17	(Z)-β-ocimeme	MH	1038	1032	-	-	0.2 ± 0.02	0.2 ± 0.05	-	-	-
18	(*E*)-β-ocimeme	MH	1049	1044	-	-	19.8 ± 0.25	2.4 ± 0.78	0.3 ± 0.28	-	-
19	γ-terpinene	MH	1060	1054	13.3 ± 3.08	-	-	1.0 ± 0.48	-	-	6.0 ± 0.34
20	cis-sabinene hydrate	OM	1070	1065	0.3 ± 0.27	-	-	0.2 ± 0.20	-	-	-
21	1-octanol	NT	1071	1063	-	-	-	-	0.1 ± 0.08	-	-
22	terpinolene	MH	1088	1086	-	-	-	0.6 ± 0.06	-	-	-
*23*	benzoic acid, methyl ester	NT	1094	1091 $	0.2 ± 0.17	-	-	-	-	-	-
24	linalool	OM	1099	1095	17.1 ± 0.92	-	-	13.7 ± 0.75	1.6 ± 0.10	0.3 ± 0.07	-
25	*n*-nonanal	NT	1100	1100	0.2 ± 0.09	-	-	-	-	-	0.6 ± 0.10
26	(*E*)-myroxide	OM	1141	1140	-	-	-	0.7 ± 0.27	0.4 ± 0.05	-	-
27	camphor	OM	1145	1141	-	-	-	1.7 ± 0.68	-	0.4 ± 0.08	6.5 ± 0.28
*28*	borneol	OM	1167	1165	-	-	-	0.3 ± 0.14	-	-	-
29	isoneral	OM	1170	1175 $	-	-	-	-	0.1 ± 0.02	-	-
30	terpinen-4-ol	OM	1177	1174	-	-	-	2.5 ± 0.20	-	-	-
31	isogeranial	OM	1185	1184 $	-	-	-	-	0.3 ± 0.05	-	-
32	α-terpineol	OM	1189	1186	-	-	-	0.1 ± 0.06	-	-	-
33	3,7-octadiene-2,6-diol,2,6-dimethyl-	OM	1190	1189 $	-	-	-	0.1 ± 0.06	-	-	-
34	methyl salicylate	NT	1192	1190	0.4 ± 0.03	-	-	-	-	-	-
35	*n*-decanal	NT	1206	1201	1.9 ± 0.35	-	-	-	-	0.5 ± 0.04	0.7 ± 0.11
36	ethanol, 2-phenoxy-	NT	1226	1221 $	0.1 ± 0.10	-	-	-	-	-	-
37	nerol	OM	1228	1227	-	-	-	-	1.8 ± 0.44	-	-
38	6-octenol, 7-methyl-3-methylene-	NT	1229	1221	-	-	-	-	0.1 ± 0.09	-	-
39	thymol methyl ether	OM	1235	1232	19.9 ± 1.45	-	-	-	-	-	-
40	β-citral	OM	1240	1235	-	-	-	-	5.5 ± 0.53	-	-
41	geraniol	OM	1255	1249	-	-	-	-	1.4 ± 0.32	-	-
42	chavicol	PP	1256	1247	-	-	0.2 ± 0.07	-	-	-	-
43	α-citral	OM	1270	1264	-	-	-	-	9.2 ± 0.34	-	-
44	bornyl acetate	OM	1285	1284	-	-	-	0.9 ± 0.03	-	-	-
45	*iso*bornyl acetate	OM	1286	1283	-	-	-	-	-	-	14.3 ± 1.66
46	thymol	OM	1292	1289	19.4 ± 1.59	-	-	-	-	-	-
*47*	carvacrol	OM	1299	1298	0.6 ± 0.10	-	-	-	-	-	-
48	tridecane	NT	1300	1300	-	-	-	-	0.1 ± 0.10	-	-
49	elemene isomer	SH	1344	1343 $	-	-	-	0.1 ± 0.07	-	-	-
50	α-cubebene	SH	1351	1345	-	-	-	0.4 ± 0.01	0.2 ± 0.01	-	-
51	eugenol	PP	1357	1356	-	-	6.9 ± 1.80	3.4 ± 1.14	-	-	-
52	neryl acetate	OM	1364	1359	-	-	-	-	0.1 ± 0.08	-	-
53	α-copaene	SH	1376	1374	0.1 ± 0.05	-	0.4 ± 0.01	1.8 ± 0.10	2.6 ± 0.13	-	6.3 ± 1.02
54	*cis*-*trans*-nepetalactone	OM	1377	1386	-	64.2 ± 0.47	-	-	-	-	-
55	β-bourbonene	SH	1384	1387	-	-	-	-	0.1 ± 0.06	-	-
56	β-cubebene	SH	1385	1387	-	-	-	0.1 ± 0.02	0.1 ± 0.03	-	-
*57*	β-cubebene	SH	1389	1387	-	-	0.6 ± 0.16	0.4 ± 0.09	1.2 ± 0.23	-	-
58	β-elemene	SH	1391	1389	0.3 ± 0.08	0.4 ± 0.15	0.1 ± 0.04	16.8 ± 1.69	0.2 ± 0.02	5.7 ± 0.43	-
59	sesquithujene	SH	1402	1405	-	-	0.2 ± 0.03	-	0.1 ± 0.03	-	-
60	α-gurjunene	SH	1409	1409	-	-	-	0.1 ± 0.06	-	-	-
61	isodihydronepetalactone	OM	1413	1414 §	-	0.3 ± 0.13	-	-	-	-	-
62	β-caryophyllene	SH	1419	1417	3.1 ± 1.15	19.0 ± 1.17	4.6 ± 0.19	2.5 ± 1.03	23.7 ± 2.00	36.2 ± 7.93	2.2 ± 0.27
63	β-copaene	SH	1432	1430	-	0.2 ± 0.03	0.4 ± 0.25	1.1 ± 0.84	0.7 ± 0.41	-	-
64	β-gurjunene	SH	1434	1431	-	-	-	0.1 ± 0.08	-	-	-
65	*cis*-geranylacetone	AC	1435	1445 $	-	-	-	-	-	-	0.6 ± 0.08
66	*trans*-α-bergamotene	SH	1435	1432	-	-	6.4 ± 0.03	-	11.6 ± 0.57	-	-
67	α-guaiene	SH	1439	1437	-	-	-	9.0 ± 0.03	-	-	-
68	(*Z*)-β-farnesene	SH	1444	1440	-	-	1.4 ± 0.10	-	-	-	-
69	*iso*germacrene D	SH	1448	1446 §	-	-	-	-	0.8 ± 0.11	-	-
70	trans-geranylacetone	AC	1453	1452 $	0.2 ± 0.19	-	-	-	-	-	-
71	*cis*-muurola-3,5-diene	SH	1454	1448	-	-	-	1.0 ± 0.40	-	-	-
72	α-humulene	SH	1455	1452	-	0.8 ± 0.11	1.9 ± 0.05	2.1 ± 0.31	3.2 ± 0.36	6.0 ± 0.93	-
73	(*E*)-β-famesene	SH	1457	1454	-	0.5 ± 0.23	2.5 ± 0.11	-	0.3 ± 0.02	0.7 ± 0.21	-
74	*cis*-muurola-4(14),5-diene	SH	1463	1465	-	-	0.3 ± 0.11	1.4 ± 0.18	0.5 ± 0.04	-	-
75	γ-muurolene	SH	1477	1478	-	-	0.1 ± 0.07	0.3 ± 0.05	0.3 ± 0.04	-	0.9 ± 0.39
76	germacrene D	SH	1481	1484	6.7 ± 0.73	8.0 ± 2.13	8.4 ± 1.25	17.3 ± 1.07	13.4 ± 1.35	1.7 ± 0.70	-
77	2-isopropenyl-4a,8-dimethyl-1,2,3,4,4a,5,6,7-octahydronaphtalene	SH	1485	1485 $	-	-	-	0.4 ± 0.09	-	-	-
78	β-selinene	SH	1486	1489	-	-	-	0.3 ± 0.03	-	0.7 ± 0.09	-
*79*	bicyclosesquiphellandrene	SH	1489	1488 $	-	-	-	0.1 ± 0.04	0.2 ± 0.03	-	-
80	bicyclo[7.2.0undec-4-ene,4,11,11-trimethyl-8-methylene-	NT	1490	1504 $	-	-	0.8 ± 0.06	-	1.1 ± 0.06	-	-
81	(*Z,E*)-α-farnesene	SH	1491	1498 $	-	-	-	-	-	0.8 ± 0.07	-
82	cis-muurola-4(14),5-diene	SH	1492	1491 §	-	-	-	-	0.2 ± 0.04	-	-
83	valencene	SH	1493	1496	-	-	-	-	-	-	-
84	*epi*-cubebol	OS	1493	1493	-	-	-	-	-	-	1.5 ± 0.19
85	α-zingiberene	SH	1495	1493	-	1.0 ± 0.25	-	-	-	0.9 ± 0.09	-
86	γ-amorphene	SH	1496	1495	-	-	-	-	0.1 ± 0.10	-	-
87	aciphyllene	SH	1499	1501	-	-	-	1.1 ± 0.19	-	-	-
88	β-bulnesene	SH	1505	1508 $	-	-	-	9.5 ± 0.68	-	-	-
89	cis-α-bisabolene	SH	1507	1506	-	-	0.1 ± 0.00	-	-	-	-
90	β-bisabolene	SH	1509	1505	-	0.5 ± 0.16	26.2 ± 1.56	-	0.9 ± 0.06	4.0 ± 1.60	-
91	γ-cadinene	SH	1513	1513	-	0.2 ± 0.01	-	2.9 ± 0.33	0.6 ± 0.09	-	-
92	cubebol	OS	1515	1514	-	-	-	-	-	0.5 ± 0.20	3.0 ± 0.50
93	β-sesquiphellandrene	SH	1524	1521	-	-	-	-	-	1.3 ± 0.02	-
*94*	δ-cadinene	SH	1525	1522	-	0.5 ± 0.05	0.6 ± 0.10	0.8 ± 0.01	1.3 ± 0.10	-	5.3 ± 0.55
*95*	trans-γ-bisabolene	SH	1533	1531 $		0.2 ± 0.08	-	-	-	-	-
*96*	α-cadinene	SH	1538	1537	-	-	0.1 ± 0.06	0.2 ± 0.03	0.2 ± 0.04	-	-
*97*	*trans*-α-bisabolene	SH	1545	1545 $	-	-	17.3 ± 2.00	-	15.4 ± 0.47	-	-
98	elemol	OS	1549	1548	-	-	-	-	-	1.1 ± 0.28	-
99	guaiol	OS	1596	1600	-	-	-	-	-	0.2 ± 0.04	11.5 ± 0.41
100	10-epi-γ-eudesmol	OS	1619	1622	-	-	-	-	-	-	0.4 ± 0.04
101	τ-cadinol	OS	1640	1638	-	-	-	0.2 ± 0.01	-	-	-
102	β-eudesmol	OS	1649	1649	-	-	-	-	-	-	1.1 ± 0.17
103	Methyl dihydrojasmonate	NT	1650	1648 §	-	-	-	-	-	0.2 ± 0.20	-
104	α-eudesmol	OS	1653	1652	-	-	-	-	-	-	2.7 ± 0.27
105	(+)-valeranone	OS	1677	1674	-	-	-	-	-	-	1.0 ± 0.13
106	elemol acetate	OS	1679	1680	-	-	-	-	-	9.0 ± 1.87	-
107	(E)-α-santalol	OS	1680	1687 $	-	-	-	0.1 ± 0.10	-	-	-
108	β-bisabolol	OS	1684	1674	-	-	-	-	-	1.0 ± 0.17	-
109	2,2,6-trimethyl-1-(3-methylbuta-1,3-dienyl)-7-oxabicyclo[4.1.0] heptan-3-ol	NT	1692	1692 $	-	-	-	0.2 ± 0.18	-	-	-
110	β-sinensal	NT	1695	1700	-	0.1 ± 0.10	-	-	-	-	-
111	benzyl benzoate	NT	1762	1759	0.2 ± 0.20	-	-	-	-	-	-
112	α-sinensal	OS	1752	1755	-	-	-	-	-	0.7 ± 0.25	-
113	hexahydrofarnesyl acetone	AC	1844	1845 $	0.1 ± 0.10	-	-	-	-	-	-
114	pentylcurcumene	NT	1950	1951 $	-	0.1 ± 0.10	-	-	-	-	-
115	3,7,11,15-tetramethyl-2-hexadecen-1-ol	NT	2116	2116 &	-	-	-	-	-	2.5 ± 0.12	-
116	sandaracopimarinol	OD	2279	2269	-	-	-	-	-	2.2 ± 0.44	-
117	communic acid	NT	2405	2365	-	-	-	-	-	3.6 ± 0.65	-
118	methyl neoabietate	OD	2435	2443	-	-	-	-	-	6.3 ± 0.89	-
	Number of identified peaks				21	16	24	51	38	27	21
	Class of Compounds				M. did.	N × faas	Ob-BS	Ob-Cn	Ob-Ct	S. disc	S. micro
	Monoterpene hydrocarbons (MH)				29.0 ± 4.71	2.3 ± 0.28	20.0 ± 0.50	5.3 ± 0.44	-	2.4 ± 0.34	36.8 ± 5.91
	Oxygenated monoterpenes (OM)				57.3 ± 4.32	66.8 ± 1.35	-	20.8 ± 0.21	20.4 ± 1.52	0.7 ± 0.13	25.6 ± 3.83
	Sesquiterpene hydrocarbons (SH)				10.2 ± 1.55	31.3 ± 2.03	71.6 ± 0.08	69.8 ± 4.06	77.9 ± 2.21	58.0 ± 8.11	14.7 ± 0.97
	Oxygenated sesquiterpenes (OS)				-	-	-	0.3 ± 0.16	-	12.5 ± 2.61	21.2 ± 0.89
	Oxygenated diterpenes (OD)				-	-	-	-	-	8.5 ± 0.45	-
	Phenylpropanoids (PP)				-	-	7.1 ± 1.87	3.4 ± 1.14	-	-	-
	Apocarotenoids (AC)				0.3 ± 0.05	-	-	-	-	-	-
	Non-terpene derivatives (NT)				3.0 ± 0.32	0.2 ± 0.03	1.3±0.59	0.3 ± 0.06	1.4 ± 0.21	7.9 ± 1.27	1.3 ± 0.38
	Total Identified (%)				99.8 ± 0.20	98.5 ± 0.50	100 ± 0.00	99.9 ± 0.01	100 ± 0.00	90.0 ± 4.41	99.6 ± 0.53

^1^ value in tables are the mean of 3 triplicates; * Standard deviation; RI (exp): relative retention index determined on HP-5MS capillary column; RI (lit) relative retention index from Adams (1996); §: relative retention index found in pherobase.com; $: relative retention index found in NIST 2014; &: relative index found in pubchem (pubchem.ncbi.nlm.nih.gov).

**Table 4:** Chemical composition of the flower EOs from the studied Lamiaceae species (n = 3) ^1^.

	Compounds	Class	RI (exp)	RI (lit)	M. did	N. × faas	Ob-BS	Ob-Cn	Ob-Ct	S. disco	S. micro
					Relative abudance (%)						
1	5,5-dimethyl-2(5H)-furanone	nt	952	952	-	-	-	-	-	-	2.3 ± 0.38
2	eucalyptol	om	1032	1026	-	0.3 ± 0.09 *	-	-	-	-	-
3	3,5-octadien-2-ol	nt	1038	1037	-	-	-	-	-	-	1.2 ± 0.61
4	*cis*-sabinene hydrate	om	1070	1068	0.6 ± 0.06	-	-	-	-	-	-
5	linalool	om	1099	1095	10.2 ± 1.12	-	-	48.6 ± 1.64	1.4 ± 0.06	-	1.3 ± 0.90
6	terpinen-4-ol	om	1177	1074	-	-	-	23.7 ± 1.90	-	-	2.3 ± 0.12
7	isocreosol	pp	1201	1202	-	0.9 ± 0.32	-	-	-	-	-
8	nordavanone	om	1230	1234	-	-	-	-	-	-	2.4 ± 0.76
9	pulegone	om	1237	1237	-	0.1 ± 0.08	-	-	-	-	-
10	β-citral	om	1240	1245	-	-	-	-	18.8 ± 0.90	-	-
11	camphor	om	1245	1143	-	-	-	-	-	-	1.4 ± 0.41
12	α-citral	om	1270	1271	-	-	-	-	32.2 ± 1.68	-	-
13	benzenepropanoic acid, methyl ester	nt	1279	1280	-	0.3 ± 0.09	-	-	-	-	-
14	isobornyl acetate	om	1286	1290	-	-	-	-	-	-	0.6 ± 0.14
15	thymol	om	1291	1289	68.6 ± 3.43	0.4 ± 1.15	-	-	-	-	8.1 ± 0.95
16	carvacrol	om	1299	1298	4.5 ± 0.99	-	-	-	-	-	10.9 ± 1.95
17	eugenol	pp	1357	1356	-	-	17.6 ± 0.25	10.7 ± 0.43	-	-	1.6 ± 0.76
18	*cis-trans*-nepetalactone	om	1377	1393	-	0.8 ± 0.22	-	-	-	-	-
19	β-bourbonene	sh	1384	1385	-	0.3 ± 0.04	-	-	-	-	-
20	β-caryophyllene	sh	1419	1417	-	2.4 ± 0.26	-	-	-	1.2 ± 0.61	-
21	α-bergamotene	sh	1435	1438	-	-	0.5 ± 0.01	-	-	-	-
22	2,6,10-trimethyltridecane	nt	1449	1461	-	-	-	-	-	0.2 ± 0.11	-
23	α-humulene	sh	1454	1452	-	0.4 ± 0.04	-	-	-	0.3 ± 0.16	-
24	(*E*)-β-famesene	sh	1457	1454	-	0.4 ± 0.05	0.4 ± 0.02	-	-	-	-
25	germacrene D	sh	1481	1484	-	-	3.4 ± 0.04	-	-	2.3 ± 0.51	-
26	α-curcumene	sh	1483	1486	-	0.8 ± 0.04	-	-	-	-	-
27	1-(3,6,6-trimethyl-1,6,7,7a-tetrahydrocyclopenta[c]pyran-1-yl) ethanone	nt	1484	-	-	0.3 ± 0.07	-	-	-	-	-
28	bicyclosesquiphellandrene	sh	1489	1488	-	-	-	-	3.3 ± 0.21	-	-
29	davana ether	os	1490	1491	-	-	-	-	-	-	16.3 ± 1.51
30	α-farnesene	sh	1508	1509	-	-	-	-	-	0.2 ± 0.06	-
31	β-bisabolene	sh	1509	1505	-	0.8 ± 0.02	34.4 ± 2.02	-	-	-	-
32	*trans*-α-bisabolene	sh	1512	1545 $	-	-	38.7 ± 2.94	-	29.3 ± 0.80	-	-
33	γ-cadinene	sh	1513	1511	-	0.3 ± 0.00	-	-	-	-	-
34	β-sesquiphellandrene	sh	1524	1521	-	0.4 ± 0.01	-	-	-	-	-
35	cyclohexanemethanol, 4-ethenyl-α,α,4-trimethyl-3-(1-methylethenyl)-, acetate, [1R-(1α,3α,4β)]-	nt	1569	1562	-	-	-	-	-	1.5 ± 0.03	-
36	*cis*-eudesm-6-en-11-ol	os	1571	1575	-	-	-	-	-	-	4.1 ± 0.15
37	caryophyllene oxide	os	1581	1583	-	17.2 ± 1.19	-	-	-	0.1 ± 0.03	1.1 ± 0.51
38	davanone	os	1588	1586	-	-	-	-	-	-	2.8 ± 0.19
39	guaiol	os	1596	1597	-	-	-	-	-	-	4.0 ± 0.65
40	humulene epoxide II	os	1606	1608	-	1.1 ± 0.03	-	-	-	-	-
41	zingiberenol	os	1616	1620	-	1.1 ± 0.19	-	-	-	-	-
42	dill apiole	os	1622	1625	-	3.0 ± 0.31	-	-	-	-	-
43	13-nor-valenc-1(10)-en-11-one	os	1629	1628	-	-	-	-	-		3.1 ± 0.75
44	selin-6-en-4α-ol	os	1636	1636	-	0.2 ± 0.04	-	-	-	-	-
45	tau.-cadinol	os	1640	1640	-	1.6 ± 0.19	-	13.8 ± 2.94	-	-	-
46	cubenol	os	1642	1643	-	0.4 ± 0.06	-	-	-	-	-
47	10,10-dimethyl-2,6-dimethylenebicyclo[7.2.0] undecan-5β-ol	os	1644	1644	-	1.2 ± 0.11	-	-	-	-	-
48	β-eudesmol	os	1649	1651	-	-	-	-	-	-	2.0 ± 0.13
49	α-eudesmol	os	1653	1652	-	-	-	-	-	-	2.8 ± 0.41
50	precocene II	pp	1658	1659	-	2.3 ± 0.30	-	-	-	-	-
51	aromadendrene oxide-(2)	os	1678	1678	-	2.0 ± 0.08	-	-	-	-	-
52	α-bisabolol	os	1684	1683	-	-	1.3 ± 0.07	-		-	-
53	β-sinensal	os	1695	1704	-	4.1 ± 0.91	-	-	-	-	-
54	germacra-4(15),5,10(14)-trien-1β-ol	os	1695	1686 $	-	0.6 ± 0.13	-	-	-	-	3.5 ± 0.36
55	heptadecane	nt	1700	1700	-	0.6 ± 0.25	-	-	-	0.2 ± 0.04	-
56	Z-α-trans-bergamotol	os	1701	1708	-	0.1 ± 0.00	-	-	-	-	-
57	longifolenaldehyde	os	1707	1708	-	0.5 ± 0.07	-	-	-	-	-
58	cuprenenol	os	1709	1702	-	-	-	-	-	-	2.0 ± 0.06
59	β-santalol	os	1715	1720	-	-			12.0 ± 1.29	-	-
60	*cis*-nuciferol	pp	1735	1730	-	2.2 ± 0.55	-	-	-	-	-
61	(6R,7R)-bisabolone	os	1747	1737	-	1.2 ± 0.39	-	-	-	-	-
62	*cis*-lanceol	os	1763	1761	-	4.6 ± 1.57	-	-	-	-	-
63	costol	os	1778	1774	-	0.4 ± 0.15	-	-	-	-	-
64	hexadecanal	nt	1817	1818	-	-	-	-	-	0.1 ± 0.08	-
65	(*E,E*)-farnesyl acetate	os	1843	1843	-	-	-	-	-	-	4.4 ± 1.15
66	hexahydrofarnesyl acetone	ac	1844	1845	1.3 ± 0.77	1.6 ± 0.69	-	-	-	15.7 ± 2.04	11.9 ± 1.12
67	2,6,10,15-tetramethyl-benzoic acid, 2-phenylethyl ester	nt	1856	1860	-	0.1 ± 0.08	-	-	-	-	-
68	3-methyl-nonadecane	nt	1970	1972	-	-	-	-	-	0.2 ± 0.04	-
69	octadecanal	nt	2021	2021	-	-	-	-	-	0.2 ± 0.03	-
70	2,6,10,14-tetramethyl-7-(3-methylpent-4-enylidene) pentadecane	nt	2071	2068	-	-	-	-	-	-	5.3 ± 0.88
71	heneicosane	nt	2100	2100	-	0.5 ± 0.35	-	-	-	1.5 ± 0.26	-
72	phytol	od	2114	2122	-	0.3 ± 0.04	-	-	-	0.3 ± 0.06	2.5 ± 0.65
73	1 N-phenyl-naphthalenamine	nt-N	2135	2135	-	1.2 ± 0.80	-	-	-	-	-
74	3-methyl-heneicosane	nt	2171	2172	-	-	-	-	-	9.9 ± 1.75	-
75	docosane	nt	2200	2200	-	-	-	-	-	0.4 ± 0.11	-
76	eicosanal	nt	2224	2224	-	-	-	-	-	0.4 ± 0.14	-
77	sclareol	od	2227	2225	-	0.3 ± 0.14	-	-	-	-	-
78	4-methyldocosane	nt	2257	2258	-	0.9 ± 0.07	-	-	-	0.6 ± 0.21	-
79	larixol	od	2264	2265	-	0.1 ± 0.03	-	-	-	-	-
80	kolavenol	od	2297	2297	-	-	-	-	-	0.3 ± 0.12	-
81	carbonic acid, octadecyl vinyl ester	nt	2299	2299 $	-	1.4 ± 0.47	-	-	-	-	-
82	tricosane	nt	2300	2300	-	1.3 ± 0.90	-	-	-	5.0 ± 1.24	-
93	2-methyl-tricosane	nt	2363	2365	-	0.7 ± 0.48	-	-	-	4.3 ± 1.52	-
84	1-heneicosanol	nt	2380	2365	0.5 ± 0.07	-	-	-	-	-	-
85	tetracosane	nt	2400	2400	4.5 ± 0.83	14.7 ± 3.63	3.7 ± 0.53	-	-	24.3 ± 1.87	-
86	undec-10-ynoic acid, dodecyl ester	nt	2409	2409 $	-	0.3 ± 0.04	-	-	-	-	-
87	docosanal	nt	2430	2430	-	-	-	-	-	0.4 ± 0.05	-
88	2-methyltetracosane	nt	2462	2456	-	0.2 ± 0.09	-	-	-	-	-
89	(*Z*)-13-docosen-1-ol	nt	2467	2466	-	-	-	-	-	0.2 ± 0.06	-
90	retinol	od	2473	2473 $	-	1.9 ± 0.45	-	-	-	-	-
91	retinal	od	2486	2486 $	-	0.2 ± 0.03	-	-	-	-	-
92	behenic alcohol	nt	2493	2501	-	-	-	-	-	3.1 ± 0.95	-
93	pentacosane	nt	2500	2500	6.8 ± 1.42	6.8 ± 1.46	-	-	-	14.6 ± 2.76	-
94	docosyl *iso*propyl ether	nt	2524	-	-	-	-	-	-	10.2 ± 0.71	-
95	2-methylhexacosane	nt	2661	2663	-	6.9 ± 0.87	-	-	-	0.2 ± 0.03	-
	Number of identified peaks				8	52	8	4	6	28	24
	Class of compounds				M. did.	N × faas	Ob-BS	Ob-Cn	Ob-Ct	S. disco	S. micro
	Oxygenated monoterpenes (om)				83.9 ± 2.49	1.6 ± 0.42	-	72.3 ± 3.11	52.4 ± 2.51	-	27.0 ± 1.84
	Sesquiterpene hydrocarbons (sh)				-	5.8 ± 0.43	77.4 ± 2.96	-	32.6 ± 1.00	4.0 ± 1.87	-
	Oxygenated sesquiterpenes (os)				-	35.2 ± 2.29	1.3 ± 0.07	13.8 ± 1.94	12.0 ± 1.29	0.1 ± 0.03	46.1 ± 3.98
	Oxygenated diterpenes (od)				-	2.8 ± 0.84	-	-	-	0.6 ± 0.18	2.5 ± 0.56
	Apocarotenoides (ac)				1.3 ± 0.77	1.6 ± 0.69	-	-	-	15.7 ± 2.04	11.9 ± 1.12
	Non-terpenes derivatives (nt)				11.8 ± 1.02	41.2 ± 4.11	3.7 ± 0.53	-	-	77.5 ± 3.87	8.8 ± 1.87
	Phenylpropanoids (pp)				-	4.5 ± 0.85	17.6 ± 0.25	10.7 ± 0.43	-	-	1.6 ± 0.76
	Total Identified (%)				97.0 ± 2.31	92.7 ± 3.3	100 ± 0.00	96.8 ± 0.22	97.0 ± 0.17	97.9 ± 0.01	97.9 ± 0.63

^1^ value in tables are the mean of 3 triplicates; * Standard deviation; RI (exp): relative retention index determined on HP5MS capillary column; RI (lit) relative retention index from ADAMS (1996).
